# Cancer Stem Cell and Gastrointestinal Cancer: Current Status, Targeted Therapy and Future Implications

**DOI:** 10.4172/2167-0501.1000202

**Published:** 2016-02-26

**Authors:** Rizwan Ahmad, Punita Dhawan, Amar B Singh

**Affiliations:** 1 Department of Biochemistry and Molecular Biology, University of Nebraska Medical Center, USA; 2 Fred and Pamela Buffett Cancer Center, University of Nebraska Medical Center, Omaha, NE, USA; 3 VA Nebraska- Western Iowa Health Care System, Omaha, NE, USA

**Keywords:** Carcinogenesis, Stem cell, Wnt-signaling, Tumoroids, Chemoresistance

## Abstract

The cancer stem cells (CSCs) are biologically distinct subset of rare cancer cells with inherent ability of self-renewal, de-differentiation, and capacity to initiate and maintain malignant tumor growth. Studies have further reported that CSCs prime cancer recurrence and therapy resistance. Therefore, targeting CSCs to inhibit cancer progression has become an attractive anti-cancer therapeutical strategy. Recent technical advances have provided a greater appreciation of the multistep nature of the oncogenesis and also clarified that CSC concept is not universally applicable. Irrespective, the role of the CSCs in gastrointestinal (GI) cancers, responsible for the most cancer-associated death, has been widely accepted and appreciated. However, despite the tremendous progress made in the last decade in developing markers to identify CSCs, and assays to assess tumorigenic function of CSCs, it remains an area of active investigation. In current article, we review findings related to the role and identification of CSCs in GI-cancers and discuss the crucial pathways involved in regulating CSCs populations’ development and drug resistance, and use of the tumoroid culture to test novel CSCs-targeted cancer therapies.

## Introduction

The Gastrointestinal (GI) cancer refers to the malignant conditions of the gastrointestinal tract and accessory organs of digestion, including the esophagus, stomach, liver, pancreas, small intestine, colon, rectum and anus. Together, GI-cancers are responsible for more cancer-associated deaths than any other cancer of epithelial origin in the human body [1]. According to the American Cancer Society (ACS) statistics from 2015, estimated 291150 new cases of GI-cancer are expected to be diagnosed while 149,300 GI-cancer patients are expected to die in 2015 in the United States of America [1]. Thus, the need to clearly understand molecular deregulations that initiate/promote GI-cancer is of utmost importance. In recent years, the postulation that cancers originate from a small subpopulation of cells known as cancer stem cells (CSCs) that possess the ability of self-renewal and proliferation in uncontrolled manner has gained rapid acceptance [2]. With regard to cancer stem cells, scientists at the American Association for Cancer Research. (AACR) workshop came to a consensus definition that “cells within a tumor that possess the capacity for self-renewal and can cause the heterogeneous lineage of cancer cells that constitute the tumor” [2]. In addition to their self-renewal capacity, CSCs are demonstrated to have the potential to metastasize and cause cancer recurrence [3,4]. This clonal evolution (stochastic) theory suggests that most of the cancers are driven by the CSCs probably through dysregulation of the self-renewal pathways which lead to an increase in CSCs population that may further undergo genetic or epigenetic changes to become fully transformed [5,6].

To date, CSCs have been discovered in a broad spectrum of solid tumors including GI-cancers [4,7–11]. These cells have been shown to be vital in tumor development and harbor the mutations needed to initiate a tumor [4,12]. However, how CSCs arise, where they come from or how to identify them in GI-cancers, still remains poorly understood. Published reports suggest that CSCs may be derived from differentiated mature cells, progenitor cells and/or stem cells pools that undergo the transdifferentiation processes [4,12,13]. It has also been proposed that cell fusion, chromosomal rearrangement and/or horizontal gene transfer, processes that frequently accompany the tissue repair processes, may also play important role in tumor initiation, progression and CSCs origin [3,4,7,12,13]. Also, deregulation of the key regulatory signaling pathways implicated in normal tissue homeostasis, such as TGF-β, Notch, Wnt, Hedgehog-signaling etc. are implicated in CSCs development and tumor progression [14–17].

However, acceptance of the theory of the clonal evolution of CSCs to support tumor growth has also posed challenge to clearly identify and establish markers to recognize CSCs and their tissue specificity. Thus, ongoing basic and translational research efforts are predominantly aimed at gaining increased understanding of the biology of these cells and methods of targeting them. Hence, discussion of the recent development and advancement in GI CSCs field will be helpful for providing novel insight into gastrointestinal cancer and their therapeutic modalities aimed at eradicating GI-malignancies. In accordance, in this review, we focus upon recent advances in the field of CSCs in GI-cancer, regulatory signaling mechanisms and potential therapeutic strategies.

## Cancer Stem Cell Identification

A successful CSCs targeted therapeutic modality will require accurate identification and characterization of the CSCs, and methods of differentiating them from normal stem cells (SC). This is why, pathognomonic surface markers identification of CSCs and their isolation is an area of intense research [11,18–21]. Of interest, markers for the human CSCs have most commonly been identified on their ability to form tumors in xenograft mouse model and spheroid in *in vitro* culture assay [2,8–11,20,22]. From these studies, CD133 emerged as a promising surface marker for the CSCs. Subsequently, CD133+ tumor-initiating cells were also isolated from GI-cancers [13,22–27]. Furthermore, CD44, an adhesion molecule with pleiotropic roles in cell signaling and migration, has been identified as a CSCs marker in gastric, pancreatic, hepatic and colorectal cancers [28–30]. The CSCs can be identified by expression of yet another cell surface marker, epithelial cell adhesion molecule (EpCAM), which is also expressed in normal epithelial progenitor cells [31]. Aldehyde dehydrogenase 1 (ALDH1) has further emerged as a surface marker for CSCs as ALDH1+ cells demonstrate the capacity of self-renewal and generating heterogeneous cell populations in pancreatic, gastric, liver and colorectal cancers [32–35]. Of note, ALDH1 is an enzyme from the ubiquitous aldehyde dehydrogenase family that catalyzes oxidation of the aromatic aldehydes to carboxyl acids [36,37]. Additionally, Lgr5 (leucine-rich repeat-containing G protein coupled receptor5) has been identified as a gastrointestinal tract stem cell marker [38]. Notably, the Lgr5+ CSCs have been reported in pancreatic, gastric and colorectal cancers [38–40]. Recently, Daniel and colleagues established that the zymogenic chief cells which reside at the base of the gastric glands of the corpus express Troy (a member of the tumor necrosis factor receptor superfamily). These Troy+ cells behave as multipotent cells and can generate almost all cell lineages of the stomach epithelium. Most notably, these Troy+ cells divide slowly and become active only after cytotoxic drug-induced tissue injury. Of interest, in the intestinal epithelium, Lgr5 negative enterocytes can de-differentiate and re-express Troy along with Lgr5 and contribute to intestinal carcinogenesis. Also, Octamer 4 (Oct-4), a member of the POU (Pit-Oct-Unc) family and essential transcription factor during human embryogenesis is considered an important stem cell marker. It is reported to be present in differentiated benign and malignant GI-cancers including colon, pancreas, hepatic, esophageal and gastric cancers. Yet another transcription factor Sox-2, a member of the sox (SRY-related HMG box) gene family that encodes transcription factors with a single HMG DNA-binding domain has been reported to be upregulated in various GI-cancers. Remarkably, expression of Sox-2 and Oct-4 associates with increased levels of CSC markers including ALDH1 and CD44 in multiple GI-cancers. However, this field is in continuous developmental process as new studies are identifying potential molecules that may serve as new CSC markers (Table 1), and may help identify CSCs in GI-cancers in tissue-specific manner.

## Signaling Pathways that Regulate Cancer Stem Cells

Specific and unique signaling pathways are expected to be active in CSCs as compared to other cancer cell population that lack stem cell properties. Some key signaling pathways, including Wnt/β-catenin, Hedgehog-, Notch- and TGF-β-signaling have been implicated in the maintenance of CSCs in GI-cancers [14–17].

The transforming growth factor-β (TGF-β) signaling occupies central position in the signaling networks that control growth, differentiation, survival and fate of the gastrointestinal epithelial cells [41] In a normal and healthy system, TGF-β acts as a tumor suppressor by inhibiting cell proliferation, inducing apoptosis and regulating autophagy. As tumors develop, they switch their response to the TGF-β and utilize this factor as a potent promoter of cell motility, invasion, metastasis and CSC maintenance [41,42]. Also, under tumorigenic conditions, TGF-β is a potent inducer of epithelial to mesenchymal transition (EMT) by regulating transcriptional activation of the Snail family proteins and TWIST, key regulators of the EMT program [43–45].

Accordingly, TGF-β signaling is one of the most commonly altered signaling pathways in GI cancers [46–48] and plays an important role in maintaining the CSCs in human pancreatic, liver, colorectal and gastric cancers [49]. Recently, Kim et al. reported a positive correlation between TGF-β1 and ALDH1, and a causal role in regulating colon cancer growth by promoting nuclear translocation of β-catenin [50].

The signal transducer and activator of transcription 3 (STAT3) also plays crucial roles in the regulation of the cellular processes associated with cancer growth and progression including proliferation, angiogenesis, tumor cell survival and immune function [51]. Dysregulated STAT3 has been documented in wide range of GI-cancers including colorectal cancer [52,53]. Of note, the STAT3 activation process starts with the Janus kinases (JAKs) which in turn are phosphorylated by specific cytokine/s or growth factor receptors in response to the external signals including interleukin-6 (IL-6), interferon-α (IFNα), tumor necrosis factor (TNF), epidermal growth factor (EGF), platelet-derived growth factor (PDGF), and TGF-β [54–56]. The importance of the JAK–STAT3 pathway however is not limited to cancer associated immune cells but also extends to CSCs [56]. In this regard, ALDH+/CD133+ cells in colon cancer and EpCAM+/CD133+ CSCs from hepatocellular cancer demonstrate increased activation of IL-6/STAT3 activity and causal association in CSCs niche expansion [57–59]. Recent evidence further suggest that feedback activation of STAT3-signaling plays prominent role in mediating drug resistance to a broad spectrum of anti-cancer therapies, and IL6/STAT3 pathway inhibitors can serve as effective means to eradicate CSCs [57,59].

The Wnt signaling pathway is the evolutionarily conserved signaling cascade crucial for the normal embryogenesis and regulates proliferation, survival and self-renewal of gastrointestinal epithelial cells [60]. Abnormal Wnt-signaling can result from both, genetic and epigenetic changes and is detected in variety of GI-cancers [61–63]. In recent years, this pathway has also been found to regulate stem cell biology in the gastrointestinal organs in adult life [64]. The Wnt pathway diversifies into three branches, the canonical (Wnt/β-catenin), non-canonical planer cell polarity (PCP), and the Wnt/calcium (non-canonical) [60]. The canonical pathway requires Wnt ligand binding to the Frizzled (FZD) receptor as well as low density lipoprotein receptor related protein 5/6 co-receptor (LRP5/6) to initiate intracellular signaling via β-catenin nuclear translocation. The signaling process starts when a Wnt ligand binds to the FZD receptor and induces dishevelled (DVL) phosphorylation which subsequently recruits Axin thereby deconstructing the degradation complex and thus helps in the stabilization of β-catenin and activation of the β-catenin–T cell-specific transcription factor (TCF)–lymphoid enhancer-binding factor (LEF) transactivation complex [65–67]. Without Wnt ligand binding, the cytoplasmic β-catenin is phosphorylated by a destruction complex and degraded in the proteasomes. This degradation complex is composed of the tumor suppressor adenomatous polyposis coli (APC), the scaffolding protein AXIN, CK1 (casein kinase 1) and GSK-3 (glycogen synthase kinase 3). The non-canonical Wnt-pathways generally associate with differentiation, cell polarity and migration. In the non-canonical PCP pathway, Wnt ligands bind the FZD receptor and activate small GTPases such as Ras homolog gene family member A (RhoA), Ras-related C3 botulinum toxin substrate (RAC) and cell division control protein 42 (Cdc42), via recruitment and activation of DVL. In the calcium-dependent non-canonical Wnt-signaling, Wnt ligands bind to both, the FZD receptor and alternative receptors of tyrosine kinase family also known as the RYK (receptor-like tyrosine kinase) or ROR (tyrosine kinase-like orphan receptor). This pathway boosts cell migration and inhibition of the canonical Wnt signaling via intracellular calcium flux and activation of the calmodulin kinase (CaMK2), Jun kinase (JNK) and PKCα. Of note, Notch-activation can also downregulate active β-catenin levels by post-translational regulation of the β-catenin endo-lysosomal degradation [68,69]. The “canonical” Wnt/β-catenin pathway plays a crucial role in modulating the balance between self-renewal and differentiation by β-catenin delocalization in several adult CSCs [70]. This process allows for regulation of the stem cells (SCs) and its dysfunction could lead to the expansion of CSCs. Recently, EpCAM and CD133 were identified as direct transcriptional targets of the Wnt/β-catenin signaling in hepatocellular carcinoma (HCC) [31]. Notably, knockdown of EpCAM in HCC stem cells decreased proliferation, colony formation and migration [31]. Additionally, siRNA-knockdown of β-catenin inhibits CSCs [31]. In the intestine, Wnt/β-catenin signaling activation occurs upon Apc mutation which leads to the familial adenomatous polyposis (FAP) syndrome [60]. In the majority of sporadic colorectal cancers, functional loss of the Apc gene seems to be one of the early events during carcinogenic events. Of note, severe polyposis in Apc mutant (Apc1322T) mice associates with increased expression of the stem cell markers Lgr5, Musashi1, Bmi1 and the Wnt target CD44 [71]. Furthermore, deletion of the Wnt target gene CD44 in Apcmin/+ mice attenuates intestinal tumorigenesis [72]. Overall, these studies support the cancer stem cell model in gastrointestinal tumorigenesis and the key role of the Wnt-signaling in the maintenance of CSC niche to promote cancer progression.

The Notch signaling pathway plays an important role during embryogenesis, cellular homeostasis and differentiation, and has great significance in multiple aspects of cancer biology, from CSCs to angiogenesis to tumor immunity [16,69,73–76]. In general, Notch signaling pathway is complex and multidimensional, imitating its roles in various functional activities [16,73–76]. Notch mediates a number of biological processes through four Notch receptor (Notch1–4) and five Notch ligands like as Delta-like ligand 1, 3 and 4, and Jagged1 and Jagged2 [73]. In canonical Notch-signaling, cell-to-cell contact is generally necessary for Notch activation where Notch can be cleaved through a series of proteolytic cleavages by multiple enzymes leading to the release of the active Notch fragment and activation of Notch target genes [69,73]. The Notch target genes include NF-κB, c-Myc, cyclin D1, Akt, mTOR and vascular endothelial growth factor (VEGF) [69,73,74,77,78]. Notch receptors and ligands are expressed differently among different GI-cancers. The non-canonical Notch signaling which is independent from ligand receptor interaction has also begun to be delineated and some of its functions have importance to GI-cancer malignancy [16]. Crosstalk with Wnt and/or Hedgehog (HH) pathways might also determine the overall effect of Notch signaling adding an additional layer of complexity and can serve as a tumor suppressor or oncogene in a particular tissue [15].

For instance, activation of Notch signaling can have tumor suppressor function in the HCC but may play an oncogenic role in the colon and pancreatic cancers [76]. Irrespective, Notch signaling has been found to play pivotal role in the CSCs expansion. In this regard, Notch-1 and −2 are overexpressed in pancreatic CSCs and associate with increased CD44 and EpCAM positive CSCs. Similarly, MUSASHI-1, a neural RNA binding protein and stem cell marker, found in the colonic crypts associate with regulation of the Notch signaling in the colon [76–80].

The Hedgehog (HH) signaling pathway is involved in embryonic development, repair of the normal tissues and EMT by controlling cell fate specification and pattern formation [81]. In mammals, there are three HH ligands proteins; Sonic hedgehog (SHH), Indian hedgehog (IHH), and Desert hedgehog (DHH). These proteins bind to the transmembrane receptor Patched1 (PTCH1) causing its internalization and removing its repression of the trans-membrane protein Smoothened (SMO) and thus allow pathway activity [15]. Subsequently, signaling initiated by SMO leads to activation and nuclear localization of Glioma-associated oncogene (GLI) transcription factor, which drives the expression of HH target genes including c-myc, cyclin D1, VEGF, BCL2, patched family receptor and Hairy Enhancer of Split (HES) family proteins [15]. These target genes are involved in proliferation, survival, and angiogenesis [81]. Emerging evidence from the digestive tract tumors suggest that HH signaling regulates CSCs [82]. In this regard, activated HH signaling as evidenced by relatively higher expression of the *GLI1*, *GLI2*, *PTCH1*, and Hedgehog-interacting protein (HIP) has been reported within the CSCs compartment in colorectal cancer [26]. Additionally, the target gene *SNAIL1*, which is associated with EMT and implicated in metastasis, increases in CSCs with disease progression in colorectal cancer [26,83]. In contrast, HH pathway activity inhibition by Cyclopamine or siRNA against *SMO*, *GLI1*, and *GLI2* reduced tumor cell proliferation and induced apoptosis [83]. The relationship between EMT and clonogenic growth potential has also been examined in pancreatic CSCs, and cyclopamine has been reported to inhibit each of these functional properties and the formation of metastatic disease [84]. Additionally, genes involved in the hedgehog pathway are highly expressed in CD133+ liver CSCs [85].

Recent reports further showed importance of the mTOR pathway in GI-cancer pathogenesis [86]. PIK3 (Phosphoinositide 3-kinase) is mutated in a number of cancers including gastric and colorectal cancers [87]. Many Human cancers including GI-cancers exhibit Akt activation which associates with the poor prognosis [86]. Notably, Akt1 and Akt2 overexpression has been detected in gastric, pancreatic and colorectal cancers [88,89]. The mTOR complex1 (mTORC1) and mTOR complex2 (mTORC2) are elevated in hepatic, pancreatic, gastric and colorectal cancer and regulate EMT, motility and metastasis during cancer progression [86]. Furthermore, radio-resistance in GI-cancers is associated with EMT and increased CSCs phenotypes via activation of the PI3K/Akt/mTOR signaling pathway [90,91]. Recent study on colon cancer cells further showed that PI3K/Akt/mTOR pathway inhibition suppresses colon CSCs proliferation and reduces stemness, as indicated by CD133 and Lgr5 expression [92,93]. The mTOR suppression also decreases ALDH1 activity, which is a marker for the colorectal CSCs [94]. Similarly, inhibition of mTORC2 led to a decrease in EpCAM expression in hepatic CSCs with little or no tumorigenicity in hepatocellular CSCs [95]. Using gastrointestinal tumor cells, Matsumoto et al. and Yang et al. further showed that mTOR inhibition increase the CD133+ subpopulation and trigger the conversion of CD133− to CD133+ population *in vitro* [92, 96].

Taken together, above described classical signaling pathways play crucial roles in GI-oncogenesis and CSCs self-renewal [97]. In the light of growing reports supporting the postulation that GI-cancer are diseases driven by the multipotent, self-renewing CSCs, it is critical that we understand synchronized action of these signaling pathways in regulating CSCs evolution and expansion. Such an accomplishment may lead to more effective and early diagnosis of cancer, and the development of therapeutic modalities to prevent cancer recurrence and/or therapy-resistance.

## Why Chemo and/or Radiation-Therapies Fail to Kill Cancer Stem Cells?

We know that tumors are functionally heterogeneous and only CSCs show tumorigenic capability. The conventional therapeutic strategies are mostly not able to eradicate the critical CSCs and therefore result in cancer relapse. The repeated cancer recurrence may also be due to the preferential killing of differentiated cells while leaving CSCs behind. Thus, a clear understanding of the mechanisms that underlie CSCs resistance to conventional treatments is necessary and may help formulate more effective therapies to overcome the resistance.

In this regard, several reports have shown that CSCs contain several classical mechanisms to escape the cell death from cytotoxic insults [98,99]. Elevated apoptosis resistance, drug-efflux pumps, enhanced efficiency of DNA repair, detoxification enzyme expression and relative dormancy/slow cell cycle kinetics, all include mechanisms known to be used by the CSCs in GI-cancers [98–101]. For example, CSCs demonstrate higher apoptotic threshold and elevated numbers of the ATP-binding cassette (ABC) transporter family proteins, also known as drug resistance pump [101]. Hypoxia may also lead to the radio-resistance among cancer stem cells [102], as tumors containing hypoxic cells are more radio-resistant than well-oxygenated cells. Of note, hypoxia affects stem cell generation and maintenance in GI-tumors through the expression of OCT4 (octamer-binding transcription factor 4) and c-myc activity, potentially induced by the HIF (Hypoxia-inducible factor) [103]. Both, acute and chronic hypoxia increase radio-resistance among GI-cancer cells by evading cell cycle arrest [103]. In accordance, a key outcome of the CSCs resistance to radiation and chemotherapy is selection of more resistant CSCs clonal subpopulation within a heterogeneous CSCs population [98,104,105]. In this regard, CD133^+^ CSCs were preferably enriched in chemotherapy-resistant liver, pancreatic, colorectal and gastric cancers [106–108]. Yet another study showed EpCAM^+^/CD44^+^ cell enrichment in therapy resistant gastric cancers [109]. In line, the CD133^+^ and CD44^+^ double positive CSCs cells enrichment was observed during the chemotherapy resistant CRC cell lines development [110]. Accordingly, the xenograft mouse model and *in vitro* studies demonstrated that the upregulation of Sox-2 is an important factor in chemotherapeutic drug resistance in gastric cancer cells while Oct4 upregulation associated with chemoresistance in the pancreatic, colorectal and hepatic cancer cells. Collectively, these studies indicate that cancer cell lines or primary tumor-derived cells with CSCs properties display decreased sensitivity to chemo- and radiotherapy. As expected, dysregulation of several signaling pathways including TGF-β, Wnt-, Notch-, Hedgehog-, PI3K/Akt/mTOR and EGFR etc., may play important roles in chemotherapeutic resistance in CSCs in GI-cancers [111].

Yet another potentially challenging issue in resistance to the anti-cancer therapy is the quiescent CSCs which are defined as the slow dividing CSCs [112]. Such quiescent cells are identified in different GI-cancers with various surface markers including CD133^+^, CD24^+^/CD44^+^, Lrig1 and ALDH [112]. Quiescent cancer stem cells effectively repair DNA damage and therefore survive during chemotherapy [112]. These surviving CSCs therefore can promote cancer recurrence and are associated with worst prognosis [113]. However, according to the CSCs concept, drug resistance is caused predominantly by the intrinsic or acquired resistance mechanisms among regular CSCs [113]. In the following section, we will focus upon recent CSCs-focused therapeutic approaches in cancer treatment.

## Targeting Therapies against Cancer Stem Cells

**Figure 1:**
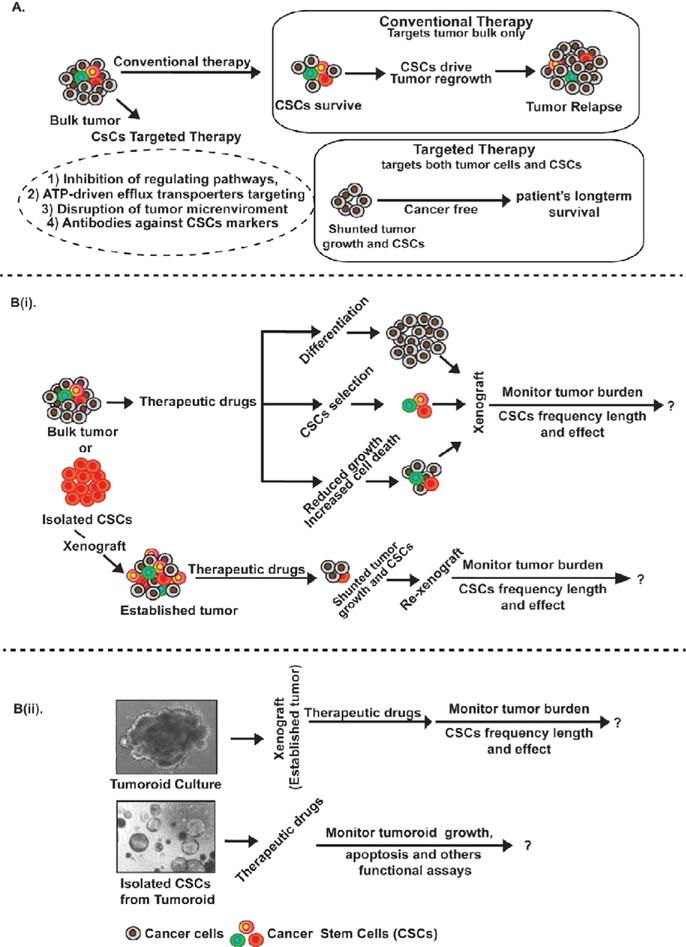
Potential therapeutic approaches and preclinical model of strategies for novel anti-CSCs therapies. A. CSC-directed therapeutic strategies may include conventional and combinational therapies; the upper box shows conventional anti-cancer therapy and how it results in cancer relapse by failing to target the CSCs. The combinational anti-CSC therapies may exert a direct cytotoxic effect on CSCs or inhibit their growth by inducing differentiation as shown in the lower box. B (i): Treatment of the tumor bulk or isolated CSCs in vitro with potential therapeutic drugs to determine their mode of action (differentiation, CSCs selection and/or decreased growth/increased cell death) or by first establishing the xenograft tumor in vivo and then evaluate efficacy of the therapeutic drugs upon tumor burden, frequency of CSCs, and durability of the effect; B (ii): Isolation of the tumors or CSCs from mouse or human to grow in in vitro 3d-culture to evaluate the efficacy of potential therapeutic drugs upon tumor growth/reduction, differentiation status, durability of effect, and ability of any residual disease to serial transplant.

Multiple novel therapeutic modalities have been designed for killing CSCs. In such an endeavor, both, surface identification marker differences and changes in signaling pathways are appealing therapeutic targets [13,114]. In accordance, scientists have designed several potential CSCs therapeutic targets which include the anti-apoptotic proteins, ABC superfamily, and transporter detoxifying enzymes, DNA repair enzymes and small molecule inhibitors to the oncogenic signaling pathways, however with varied success in effective killing of CSCs and inhibiting cancer growth [13,114] (Figure 1A).

## Targeting Key Signaling Cascades Promoting Cancer Stem Cells

The mechanisms that uphold self-renewal behavior of CSCs are also the pathways of greatest importance for the discovery and development of anticancer drugs targeting CSCs [13,113]. As described, dysregulation or over-activation of the Wnt-, Notch-, Hedgehog-, PI3K/Akt/mTOR, EGFR-signaling may play important role in the recurrence and maintenance of CSCs [15,41,55,60,61,68–70,73,74,93,113]. However, these signaling pathways also play essential role in regulating normal stem cell function. Thus, it will be important to develop CSC-selective therapies that avoid potential significant side effects caused by the inhibition of normal stem cell function.

A role for the Wnt-signaling pathways has been shown in conventional drug resistance and metastasis in variety of CSCs settings, including CRC, pancreatic and HCC [17]. At present, several types of Wnt-signaling inhibitors are under ongoing development as anticancer therapies which include agents approved by the FDA for curing other diseases before their recognition as potential Wnt-pathway inhibitors, agents in preclinical development and investigational agents in clinical studies [17]. For example, Sulindac and celecoxib previously used as nonsteroidal anti-inflammatory drugs (NSAIDs) have been found to inhibit Wnt-signaling: Sulindac targets Dishevelled (Dvl) while Celecoxib inhibits β-catenin signaling by cyclo-oxygenase (COX)-dependent and COX-independent mechanisms, and has been validated for anti-neoplastic activity in colon cancer cells [63,115]. Furthermore, Glitazone, a thiazolidinedione antidiabetic agent causes reverse β-catenin translocation to the plasma membrane [116] However, additional validation of its anticancer activity in GI-cancers addicted to Wnt-signaling is required. Similarly, Salinomycin, an antibiotic, suppress Wnt-signaling transduction and kills gastric CSCs *in vitro* [117]. In this class of inhibitors, specific molecules have shown great promise as LGK974, a Porcupine inhibitor that acetylates Wnt proteins, is being investigated in a phase I clinical trial for effects upon pancreatic and colon cancers [118]. Vantitumab, a monoclonal antibody against FZD of Wnt-signaling cascade is also currently being pursued in clinical phase I trial in pancreatic cancer and HCC [119].

As described previously, Notch-signaling pathway is a highly conserved cellular mechanism for regulating cancer stem cell homeostasis. Furthermore, activation of the Notch-signaling can upregulate several factors that in turn transmit bidirectional signals among cancer cells expressing both Notch-ligands and receptors, and also to the stroma and endothelial cells. Notch inhibition can be achieved by DAPT, a γ-secretase inhibitor routinely used in *in vitro* studies [120]. The OMP-21M18, an anti-Delta-like ligand 4 (DLL4) is further been tested for inhibiting Notch-signaling in pancreatic cancer [121]. Similarly, γ-secretase inhibitors are being used to target Notch-signaling in inhibiting multiple GI-cancer and resident CSCs however with limited success [122]. Targeting the DLL4 with monoclonal antibodies is yet another strategy to inhibit Notch signaling which has shown alluring prospects in treating solid cancers [123]. Other Notch inhibitors in the clinical pipeline include monoclonal antibodies targeting various Notch receptors, monoclonal antibodies to the γ-secretase complex component nicastrin, and soluble decoy Notch receptors that can interfere with ligand–receptor interactions [17].

As mentioned previously, Cyclopanine, a small molecule inhibitor has shown alluring potential to inhibit the Hedgehog-signaling in human pancreatic cell bearing xenograft mouse model and thus may offer a potential strategy to inhibit CSCs expansion [84]. Notably, the ALDH+ cells in pancreatic cancer cells is reduced with the use of cyclopamine *in vitro* [124]. Yet another study has shown that the cyclopamine treatment can down-regulate expressions of CD44+ and CD133+ in gemcitabine-resistant pancreatic cancer cells indicating its potential efficacy in reversing gemcitabine resistance in pancreatic cancer [84]. Similarly, self-renewing properties of the gastric CSCs decreases with cyclopamine treatment [125].

Furthermore, significant advances are made in therapeutic strategies against GI-cancer growth and progression by employing combinational therapeutical approaches including specific protein tyrosine kinase inhibitors (TKIs) and antibodies against the immune components in the tumor microenvironment to inhibit the growth or deplete CSCs population [126] (Table 1). For example, certain anti-carcinogenic agents may induce the apoptotic death and/or have differentiating effect on CSCs, and thereby may constitute the useful tools for the development of more effective cancer therapies (Figure 1(Bi)). However, besides targeting the repair machinery core, significant effort is also being made with respect to targeting the execution (cell death) machinery in cancer cells. In one of these studies, inducible caspase-9 expression was demonstrated to target colon CSCs [127]. As described, anti-apoptotic proteins are highly expressed in various cancers and especially in CSCs [128]. Therefore, targeting these anti-apoptotic proteins using small molecules like, ABT-737, a small molecule inhibitor that targets BCL2, BCLXL and BCLW, tips the apoptotic balance to a more pro-apoptotic state and reverts the resistance of colon CSCs [129]. Taken together, multiple approaches are currently being tested for their efficacy against CSCs in GI-cancers however the need of combinational therapy appears to be the paramount in the war against cancer.

## Targeting Surface Markers to Kill Cancer Stem Cells

The presence of cell surface markers allow for identification of CSCs in specific cancer type [4,7]. This is why; various groups and companies are now developing immunotoxins that can directly target such CSC markers. For example, antibodies against CD133+ CSCs conjugated to paclitaxel or cytolethal distending toxin target CD133 expressing cells and show promising result in killing CSCs *in vitro* and *in vivo* [130]. Similarly, CD133-specific oncolytic measles viruses have been developed [131]. These oncolytic viruses infect CD133 expressing cells and destroy them by lysis [131].

Furthermore, the gastric CSCs express high levels of CD44v9 which is why silencing of CD44v9 expression is being developed as a novel target for treating gastric cancer [132].In addition, antibodies against CD47 show promising effects in various cancers, such as colon and pancreatic [133]. Several antibodies delete important signals from CSCs, for example, the IL-8 receptor CXCR1 is expressed almost exclusively on CSCs and repertaxin, an inhibitor of CXCR1/2, or anti-CXCR1 treatment induces cell death in CXCR1+ colon CSCs, which appears to be mediated by inhibition of Akt-signaling [132,134]. ALDH1 activity is used as a marker for the identification of high-risk patients with pancreatic cancer [32]. Treatment with multi kinase inhibitor sorafenib and xenobiotic-processing enzyme inhibitor sulforaphane could reduce the ALDH1 activity in pancreatic cancer cells and consequently, inhibit tumor growth inhibition *in vivo*, indicating the potential for a CSC-targeting therapeutic strategy [135]. Equally, EpCAM is one of the most highly- and frequently-expressed CSCs marker, being found in pancreatic and colorectal cancer. ING1 and MT201, the anti-EpCAM antibodies, are therefore showing promising result in inhibiting tumor growth in these organs in *in vitro* or *in vivo* tumorigenicity studies [136,137]. Currently, M201 is under phase II clinical trials in cancer patients. Recently, Liao MY et al. developed yet another anti-EpCAM monoclonal antibody [138].

## ATP-Driven Efflux Transporter Targeting

Antitumor drug efflux caused by ATP-driven pump is one of the fundamental reasons for chemo-resistance in GI-cancers [139]. The increased expression of ATP-binding cassette (ABC) transporters gene family contributes to the multidrug resistance (MDR) via pumping out many anti–tumor drugs, thereby resulting in low intracellular drug concentrations. ABC transporters are membrane transporter that can pump out various structurally unrelated cytotoxic drugs at the expense of ATP hydrolysis [139]. CSCs show high expression levels of ABC transporters which play a major role in their chemo-resistance in gastrointestinal cancers [140]. Investigators have designed numerous methods to dodge and neutralize, to overcome such drug resistance. Several pharmacological agents which can interact with ABC transporters have been developed to inhibit MDR [140]. The first ABC transporter inhibitor identified was verapamil [141]. Simultaneous treatment with verapamil and anticancer drugs has displayed promising therapeutic effects [141]. Furthermore, zosuquidar (LY335979) and tariquidar (XR9576), have higher selectivity and inhibitory activity without affecting the metabolism of chemotherapeutic drugs and make it possible to overcome CSCs from the resistance [140]. Here also, certain drugs are in preclinical use; for example, difluorinated curcumin enhances the sensitivity of CD44+CD166+ colon carcinoma stem cells to the combination of 5-fluorouracil and oxaliplatin by a mechanism that involves ABCG2 downregulation [142].

## Tumor Microenvironment Targeting

Direct targeting of CSCs represents first line therapeutic strategy to combat these cells. However, other therapeutics strategies are also proposed because of the rapidly growing information on tumor microenvironment which can create a niche to foster and protect CSCs from cancer therapy. Prominent cells in tumor microenvironment are fibroblasts, myofibroblasts, adipocytes and mesenchymal stem cells, infiltrating immune cells such as macrophages and neutrophils, as well as endothelial cells that make up the walls of blood vessels that extend through the tumor [143]. CXCR4, a receptor for the stromal cell–derived factor-1 (CXCL12/SDF-1α), promotes tumor progression, angiogenesis and drug resistance. Indeed, CXCR4 expression is a prognostic marker in various GI-cancers including gastric and colon carcinomas [13,143]. CXCR4 antagonists, such as Plerixafor (AMD3100) and T14003 analogs, can damage adhesive tumor-stroma interactions and therefore render cancer stem cells vulnerable to the cytotoxic drugs [144]. The novel approach of targeting the CXCR4-CXCL12 axis is currently being explored in clinical trials as well as in mouse models of gastrointestinal cancers [143]. The development of more effective anti-cancer modalities also implicates the inhibition of the angiogenic process which is necessary for the tumor vascularization and growth. Similarly, many anti-angiogenic agents that are able to interfere with the VEGF-VEGFR transduction system, including the anti-VEGF or VEGFR antibody, VEGFR antagonists and the soluble truncated form of VEGFR have been designed and observed to effectively counteract the tumor growth in animal models *in vivo* [145].

## Novel Approach for Preclinical Evaluation of Therapies

In our view, preclinical evaluation of an effective CSCs therapy requires demonstration and this testing of the therapeutic efficacy can be accomplished in a number of ways, each representing differing levels of severity, and each more precisely reflecting clinical situations. The conventional method for evaluating the efficacy of therapy against CSCs is engraftment and cell culture models. However, these strategies may not accurately reflect *in vivo* responses to this treatment since cells adapted to culture may not mimic actual primary CSCs properties (Figure 1Bi–1Bii).

## Patient Derived Xenograft (PDX) and Tumoroids

Xenograft model or hetro-transplantation of human cancer cell lines into immunodeficient mice has served, for periods, as the major preclinical screen for the development of newer cancer therapeutics. However, current cell line-xenograft tumor preclinical models could not predict success of oncology drug development because novel therapeutics that were 97% successful in *in vivo* xenograft studies fail in clinic trials. Patient-derived Xenograft (PDX) models represent the cutting edge of cancer drug development, increasing our ability to advance novel approach for preclinical testing of new anticancer compounds *in vivo* due to the preservation of key features of human cancer, which includes invasiveness, stromal reaction, tumor vasculature and cellular diversity of human carcinomas [146]. In contrast to a cell line-xenograft tumor model, PDX tumors are established from the transplantation of fresh tumor tissue from a cancer patient into an immunodeficient mouse [146]. After surgical resection, fresh tumor is mechanically or chemically digested into small pieces and then transplanted either subcutaneous or intraperitoneally into the mice, and then passaged the xenograft tumors in NOD/SCID mice to expand the amount of tumor tissue for freezing [146]. PDX models are maintained by passaging cells directly from mouse to mouse once the tumor burden becomes too high. Tumors can be engrafted heterotopically or orthotopically. Heterotopic PDX models involve implanting tumors into the subcutaneous flank of a mouse. This method allows for easier cell transfer and precise monitoring of tumor growth and location. PDX models may be superior to the traditional cell line-xenograft models of cancer because they maintain more similarities to the parental tumors. Detailed examination of PDX mice indicate that histology and gene expression profiles are retained, along with SNPs and copy number variants [146].

Recently another novel cell culture technique has developed and allowed for the derivation of multi-cellular structures named “Organoids” and “Tumoroid” from adult organ stem cells and Tumor (especially CSCs) respectively [147–155]. These structures resemble *in vivo* organ/tumor, both in structure and developmental processes, and can be grown quickly and in relatively large quantities. Although much research has focused on developing organoids/tumoroids for tissue repair, more immediate applications include high-throughput screening for therapeutics implications, ranging from the study of cellular signaling pathways and chemo-sensitization with palliative agents to the optimization of treatment protocols in personalized medicine. In addition, gene knockout and knock-in can be performed without the complications associated with organism development. This type of *in vitro* preclinical models is allowing investigators to anticipate the pattern of clinical response and design personalized clinical trials.

## Conclusion

In summary, convincing facts have shown that CSCs display abilities for self-renewal and differentiation that are critical for cancer initiation, progression, metastasis and cancer recurrence. Presently, identification of CSCs are based on surface markers however these identification markers are not universal and more are sure to come in future. Remarkably, CSCs populations are repeatedly been refined due to the identification of new markers. Thus, question arises: how many markers will be required to be considered suitable number of CSCs identification markers for the final determination? We further don’t know with certainty whether these markers change during cancer progression. These questions remain to be addressed in future and may require dynamic follow up of the cancer cells with CSCs properties in a given PDX or tumoroid model over a long period of development. Remarkably, the CSC model is also criticized due to its inability to take into account the observed heterogeneity among GI-cancers. However, this criticism may be explained by the fact that CSCs may evolve over time and give rise to cells that are both genetically and functionally heterogeneous. While, such a postulation is well in sync with the cryptic nature of cancer, it will require a rigorous set of identification markers for authentic determination of CSCs in any tumor type, depending on the organ and developmental stage. Thus, designing the novel approaches for precise isolation, Identification and target CSCs remain an area of active investigation however holds the promise to solve current issues of therapy resistance and cancer relapse, if successful.

## Figures and Tables

**Table 1:** Gastrointestinal cancer stem cell marker and FDA approved drugs.

Cancer Type	Stem Cell Marker	Drugs	Agent Class	Target	FDA Approval	CSCs genes and Pathways
Colorectal	CD24+ CD44+ CD133+ CD166+ EpCAM+ LGR5+ OLFM4+ ASCL2+ ALDH1	Trifluridine, Tipiracil (Lonsurf ®) Ziv-aflibercept (Zaltrap®) Aflibercept (Eylea®/Zaltrap®) Regorafenib (Stivarga®) Panitumumab (Vectibix®) Cetuximab (Erbitux®) Bevacizumab (Avastin®)	Small molecules Fusion protein Fusion Protein Small molecule Monoclonal Antibody Monoclonal Antibody Monoclonal Antibody	EGFR, VGEF VEGF-A, PIGF VEGF TEK, KDR EGFR EGFR VEGF	2015 2012 2011 2012 2006 2004 2004	Notch, Nanog, Oct4, Sox2, Wnt/ β catenin, C-Myc, KLF4, Lin28,PI3K/Akt/mTOR, GATA6, IL4, IL6/STAT3, TGF- β etc.
Gastric	CD7+ CD44+ CD54+ CD90+ CD133+ NESTIN ALDH1	Ramucirumab (Cyramza®)	Monoclonal Antibody	VEGFR2	2014	Hedgehog, Notch, Wnt/ β catenin,PI3K/Akt /mTOR, IL4, IL6/STAT3, TGF- β etc.
Pancreatic	CD24+ CD44+ CD133+ EpCAM+ ESA+ ALDH+ MUSASHI- 1	Irinotecan liposome (Onivyde®) Everolimus (Afinitor®) Erlotinib (Tarceva®)	Nano-formulated Molecule Small molecule Small molecule	Topoisomerase inhibitor mTOR EGFR	2015 2011 2004	Hedgehog, Notch, Wnt/ β catenin,PI3K/Akt /mTOR,IL6/STAT3, TGF- β etc.
Liver	CD49f+ CD90+ CD133+	Sorafenib (Nexavar®)	Small molecule	PDGFRB,FLT4 , KDR, KIT, RAF1, BRAF, FLT3	2005	Wnt/ β catenin,PI3K/Akt /mTOR,IL6/STAT3, TGF- β, Nanog, Oct4, Sox2, Bmi1, SALL4
Esophageal	ALDH1, CD44, CD90	Trastuzumab (Herceptin®) Ramucirumab (Cyramza®)	Monoclonal Antibody Monoclonal Antibody	HER2 VEGF	2010 2014	TGF- β, Sox9, Bmi1, YAP1
